# miR-28-5p acts as a tumor suppressor in renal cell carcinoma for multiple antitumor effects by targeting RAP1B

**DOI:** 10.18632/oncotarget.12516

**Published:** 2016-10-07

**Authors:** Cheng Wang, Caiyun Wu, Qi Yang, Meng Ding, Jinsha Zhong, Chen-Yu Zhang, Jingping Ge, Junjun Wang, Chunni Zhang

**Affiliations:** ^1^ Department of Clinical Laboratory, Jinling Hospital, State Key Laboratory of Analytical Chemistry for Life Science, NJU Advanced Institute for Life Sciences (NAILS), School of Life Sciences, Nanjing University, Nanjing 210002, China; ^2^ State Key Laboratory of Pharmaceutical Biotechnology, Collaborative Innovation Center of Chemistry for Life Sciences, Jiangsu Engineering Research Center for MicroRNA Biology and Biotechnology, NJU Advanced Institute for Life Sciences (NAILS), School of Life Sciences, Nanjing University, Nanjing 210023, China; ^3^ Department of Urology, Jinling Hospital, Nanjing University School of Medicine, Nanjing University, Nanjing 210002, China

**Keywords:** renal cell carcinoma, miR-28-5p, RAP1B, proliferation, migration

## Abstract

The incidence and mortality rate of renal cell carcinoma (RCC) have been significantly increasing; however, the mechanisms involved in RCC development and progression are unclear. In this study, we found that miR-28-5p was decreased in RCC tumor specimens and several renal carcinoma cell lines. By using a combination of luciferase reporter assays and western blotting, we identified RAP1B, a Ras-related small GTP-binding oncoprotein implicated in a variety of tumors, as a direct target of miR-28-5p in RCC. The RAP1B protein level was increased in RCC tumor specimens and renal carcinoma cell lines, and this was inversely correlated with miR-28-5p expression. *In vitro* gain-of-function and loss-of-function studies in human renal carcinoma cell lines, demonstrated that miR-28-5p suppressed cell proliferation and migration by directly inhibiting RAP1B, and this effect was reversed by co-transfection with RAP1B. In addition, the stable overexpression of miR-28-5p inhibited tumor cell proliferation *in vivo*. This newly identified miR-28-5p/RAP1B axis provides a novel mechanism for the pathogenesis of RCC, and molecules in this axis may serve as potential biomarkers and therapeutic targets for RCC.

## INTRODUCTION

Renal cell carcinoma (RCC) represents 85% of all primary renal neoplasms and is among the top 10 most common malignancies in both men and women. In 2012, it is estimated that approximately 64,000 individuals will be diagnosed with RCC and 13,570 will die from this disease in the United States [1]. The incidence of RCC has been increasing significantly and its mortality rate has reached 40% [2]. The 5-year survival rate of RCC is approximately 55% and that of metastatic RCC is approximately 10% [1]. However, the mechanisms involved in RCC development and progression are still unclear. RCC is generally resistant to chemotherapy and radiation therapy. Surgical excision of the tumor at a localized stage remains the mainstay for curative therapy [3]. Therefore, there is an urgent need to uncover the molecular mechanisms of RCC and identify potential therapeutic targets for RCC treatment.

MicroRNAs (miRNAs), small noncoding RNAs that regulate the translation of many genes by binding to the untranslated region (3′UTR) of target mRNAs, are involved in a variety of physiological and pathological processes, in particular, cancer development. Accumulating evidence shows that miRNAs are aberrantly expressed in many types of cancers, including RCC, and some of these miRNAs function as tumor suppressor genes or oncogenes during tumor development and progression [4–9]. These differentially expressed miRNAs are important regulators of all hallmarks of cancer, including cell growth, evasion of apoptosis, tissue invasion and distant metastasis, and angiogenesis [10–16]. All these data highlight the importance of miRNAs in tumor development and provide new insights into the molecular mechanisms underlying carcinogenesis. Our previous study found that miR-28-5p was markedly decreased in sera from patients with RCC compared with that from normal controls, suggesting that miR-28-5p may be involved in the development of RCC [17]. miR-28-5p has been reported to be deceased in colorectal cancer (CRC) tissues and to inhibit colorectal cancer cell proliferation, migration and invasion *in vitro* [18]. However, the functional role and mechanism of action of miR-28-5p in RCC have not been elucidated.

RAP1B, a Ras-related small GTP-binding protein that behaves as a GTPase, has been implicated as an oncogene in a variety of tumors, including esophageal squamous cell carcinoma (ESCC), CRC, T-acute lymphoblastic leukemia, non-small cell lung carcinoma, glioma and thyroid carcinoma [19–27]. RAP1B activates multiple signaling cascades associated with tumor development and progression and is involved in cell proliferation, invasion, cell adhesion and angiogenesis [19–29]. Recently, some studies have shown that RAP1B expression is inhibited at the post- transcriptional level by some tumor suppressor miRNAs. It has been shown that RAP1B is suppressed by miR-518b in ESCC and by miR-139 and miR-100 in CRC [19, 21, 23]. However, the function and mechanisms of RAP1B in RCC have not been studied.

In this study, we explored the relationship between miR-28-5p and RCC and the potential mechanisms of miR-28-5p in RCC. We found that miR-28-5p was decreased in RCC tissues. Gain-of-function and loss-of-function studies demonstrated that miR-28-5p acted as a tumor suppressor in RCC for multiple antitumor effects by directly inhibiting RAP1B. Taken together, these results indicate that the miR-28-5p/RAP1B axis may provide further insight into the pathogenesis of RCC and may represent a potential novel therapeutic target for RCC.

## RESULTS

### Downregulation of miR-28-5p in RCC tissues and renal carcinoma cell lines

**Figure 1:**
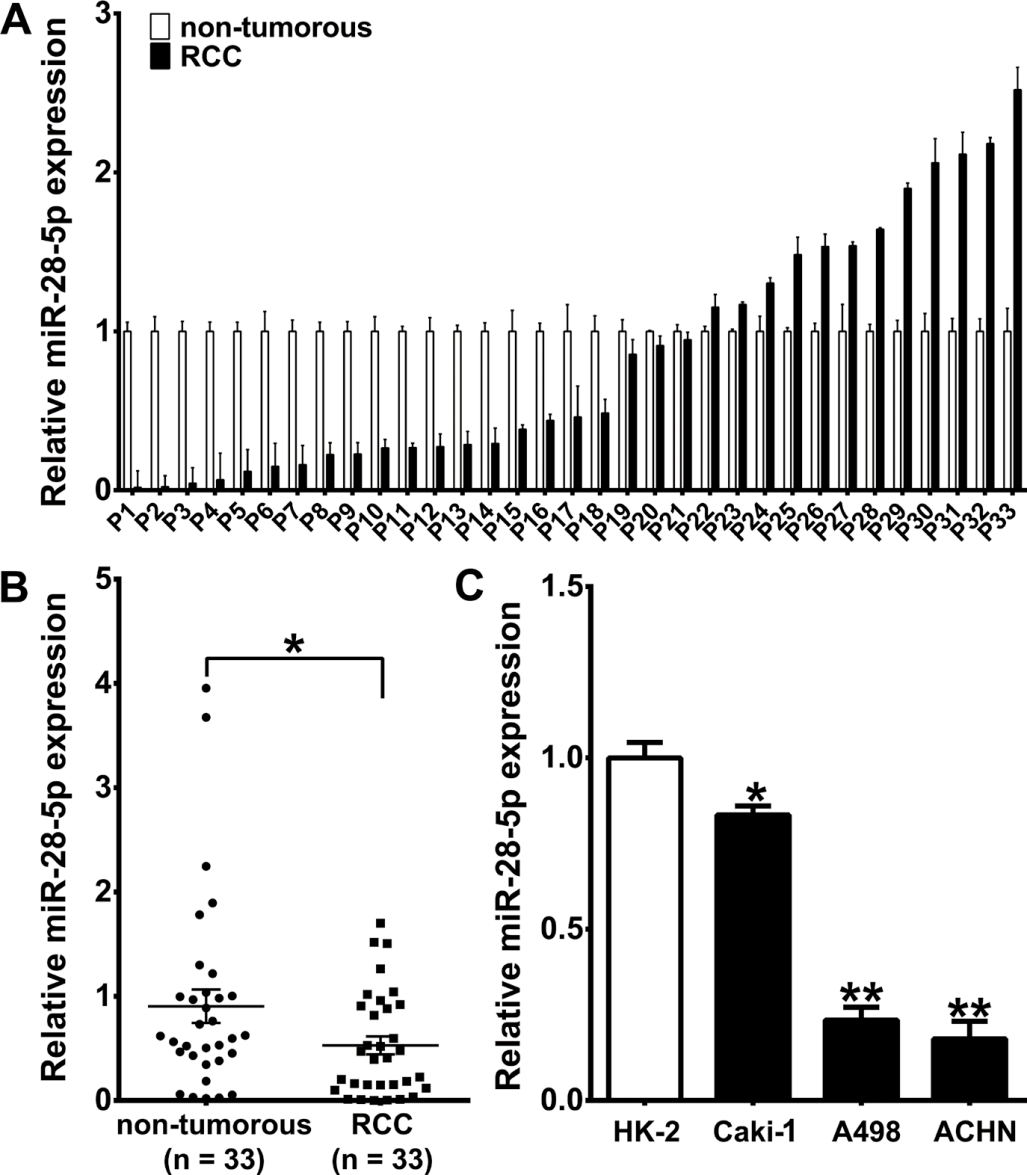
miR-28-5p expression is downregulated in RCC tissues and renal carcinoma cell lines (**A**, **B**) qRT–PCR results showed downregulation of miR-28-5p expression in 33 primary RCC samples compared with matched adjacent non- tumorous samples. The expression level of miR-28-5p was measured using qRT–PCR and normalized against an endogenous control (U6 sRNA). The data were analyzed using the ΔΔCt approach and expressed as the ration of miR-28-5p to U6 [2^–ΔCt(miR-28-5p-U6)^]. (**C**) qRT–PCR assay showed downregulation of miR-28-5p expression in three renal carcinoma cell lines. ^*^P < 0.05; ^**^P < 0.01.

To examine whether miR-28-5p was dysregulated in RCC, we first determined the expression patterns of miR-28-5p in 33 RCC tissue samples and 33 corresponding adjacent non-tumorous tissues from the same patients using real-time quantitative reverse transcriptase PCR (qRT-PCR). Most of the cancer specimens (87.9%) had the tumor histotype of clear cell RCC (Supplementary Table S1). miR-28-5p expression was deceased in 20 of the 33 (60.6%) RCC tissues when compared to the matched non-tumorous tissues (*P* < 0.05, Figure 1A and 1B). However, there was no significant association between miR-28-5p expression in RCC tissues and gender, age, histological classification or tumor-node-metastasis (TNM) stage (data not shown).

We then determined the miR-28-5p expression level in three human renal carcinoma cell lines, A498, ACHN and Caki1, and found that miR-28-5p was markedly deceased in all three cell lines compared with the immortalized primary human proximal tubular cell line HK-2 (Figure 1C). Taken together, these results from clinical specimens and cell lines indicate that the expression of miR-28-5p is significantly deceased in renal cell carcinoma.

### RAP1B is a candidate target gene of miR-28-5p

**Figure 2:**
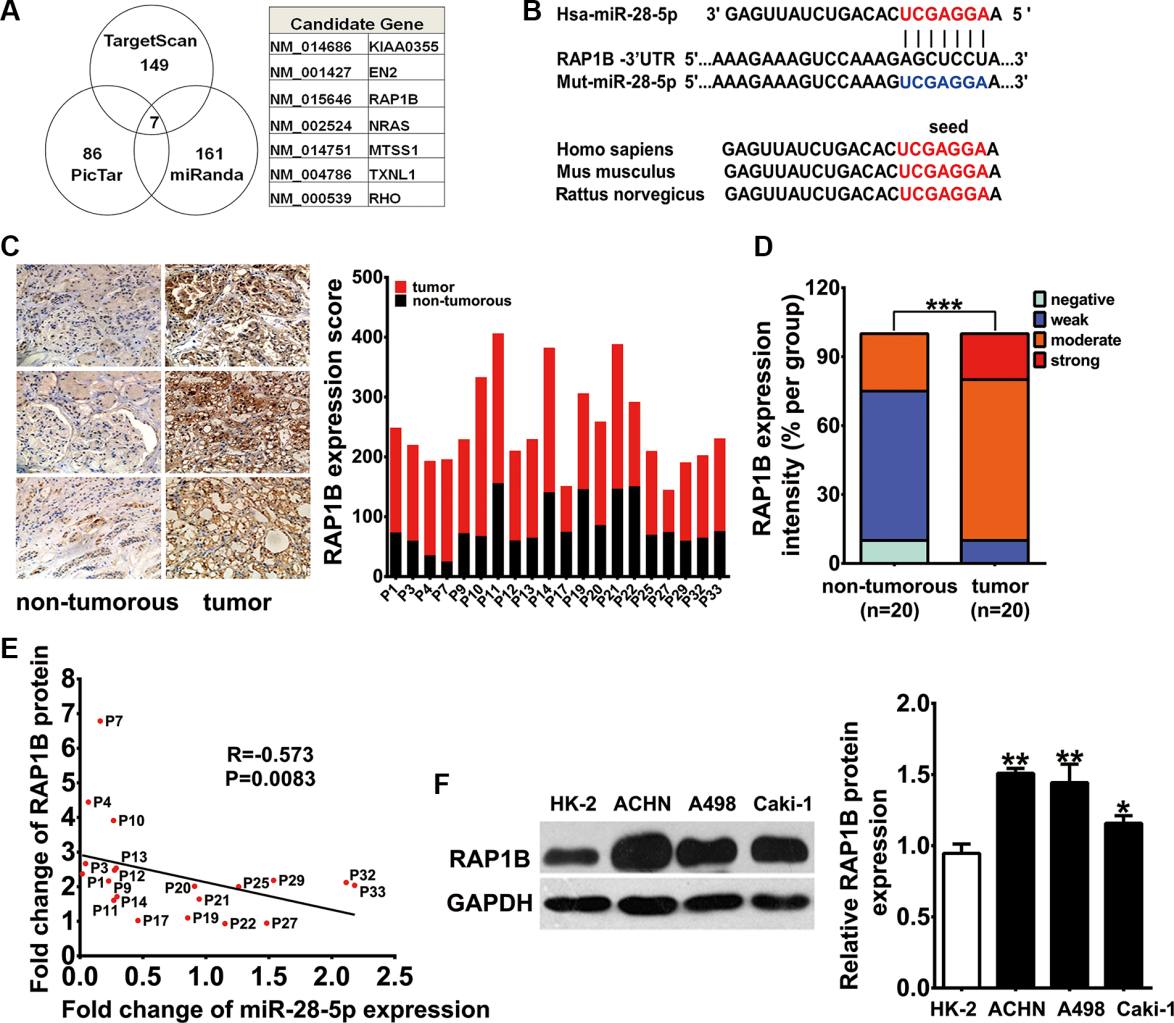
RAP1B is a downstream target gene of miR-28-5p in RCC (**A**) Schematic selection of candidate target genes with software predictive results from TargetScan, PicTar and miRanda databases. RAP1B was predicted to be a potential target gene of miR-28-5p by all three algorithms. (**B**) Schematic diagram presents the predicted miR-28-5p binding sequences and the mutated miR-28-5p binding versions. (**C**, **D**) IHC analysis showed stronger cytosolic staining for RAP1B in 20 paired RCC tissues compared with adjacent non-tumorous samples. The expression score of RAP1B in paired RCC tumor parts compared with adjacent non-tumorous tissues is shown. (**E**) By Spearman correlation analysis, a clearly inverse correlation was observed between RAP1B protein and miR-28-5p levels in the same 20 pairs of RCC tissues and adjacent noncancerous tissues (**F**) By qRT–PCR assay, RAP1B expression was upregulated in the three renal carcinoma cell lines Caki1, A498 and ACHN compared with HK-2. ^*^P < 0.05; ^**^P < 0.01.

To elucidate the mechanisms through which miR-28-5p acts on RCC, we conducted an *in silico* search using three computational algorithms in combination, TargetScan [30] (Release 6.2, June 2012, http://www.targetscan.org/vert_61/), miRanda [31] (Release August 2010, http://www.microrna.org/microrna/home.do) and PicTar [32] (Lall et al. 2006, http://pictar.mdc-berlin.de/cgi-bin/new_PicTar vertebrate.cgi), to predict the potential target genes of miR-28-5p. Computational analyses identified seven candidate genes containing potential miR-28-5p binding sites (Figure 2A). Among these candidates, RAP1B, an oncogene previously shown to play critical roles in the regulation of cell proliferation and invasion [19–25], was predicted to be a potential target of miR-28-5p (Figure 2A). One predicted hybridization was observed between miR-28-5p and the 3′-UTR of RAP1B, with perfect base-pairing between the seed region and the cognate target (Figure 2B). The minimum free energy value of the hybridizations between miR-28-5p and RAP1B was −22.7 kcal/mol, which is well within the range of genuine miRNA-target pairs. Furthermore, the miR-28-5p binding sequence in the RAP1B 3′-UTR was highly conserved across species (Figure 2B). Thus, RAP1B was selected as a candidate target gene of miR-28-5p for further validation.

### Upregulation of RAP1B protein in RCC tissues and renal carcinoma cell lines is inversely correlated with miR-28-5p

We next asked whether RAP1B was inversely correlated with miR-28-5p in human RCC tissues. We first examined the expression levels of RAP1B in 20 paired randomly selected RCC tissues and adjacent non-tumorous tissues from the 33 paired tissue samples that were used for miR-28-5p measurement by immunohistochemistry (IHC) analysis. The clinical information and the relative expression of miR-28-5p for these 20 paired tissue specimens were displayed in Supplementary Table S2. Consequently, the IHC analysis showed stronger cytosolic staining of RAP1B in RCC tissues compared with matched non-tumorous tissues (Figure 2C). RAP1B cytosolic staining was strong, moderate, and weak in 20%, 70%, and 10% of RCC tissues, respectively, while it was moderate, weak, and negative in 25%, 65%, and 10% of corresponding non-tumorous tissues, respectively (Figure 2D). The expression score of RAP1B was significantly higher in RCC tumors than in non-tumorous tissues (*P* < 0.01) (Figure 2C). More importantly, there was a clear inverse correlation between RAP1B protein and miR-28-5p levels in the same 20 pairs of RCC tissues and corresponding noncancerous tissues (R = −0.573, *P* = 0.0083) (Figure 2E).

Next, using western blotting, we found that RAP1B protein was highly expressed in another 16 paired RCC tissues and adjacent non-tumorous tissues (4 pairs were remained from the 33 paired samples and the other 12 pairs were newly collected) as well as three tested human renal carcinoma cell lines, including A498, ACHN and Caki-1, compared with HK-2 cells (Supplementary Figure S1A, S1B and Figure 2F), while no significant difference in the expression levels of RAP1B mRNA was observed between the RCC tissue samples and the corresponding adjacent non-tumorous samples (Supplementary Figure S1C). These results from clinical specimens and cell lines indicate that the expression of RAP1B protein is significantly increased in RCC.

### Validation of RAP1B as a direct target of miR-28-5p

**Figure 3:**
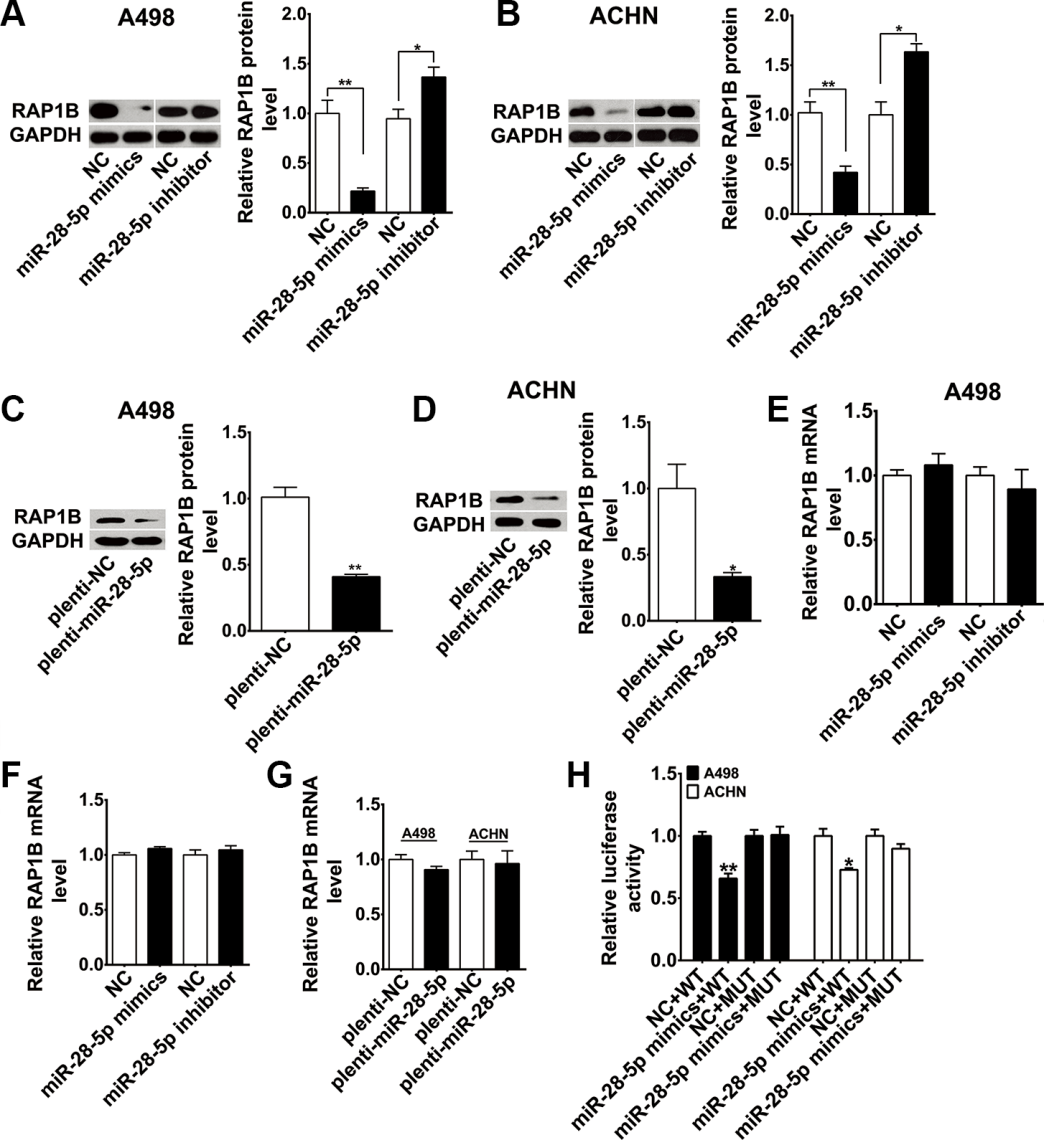
RAP1B is experimentally demonstrated as a direct target of miR-28-5p in renal carcinoma cells (**A**–**D**) By western blotting analysis, RAP1B protein was significantly downregulated in A498 and ACHN cells transfected with miR-28-5p mimics or plenti-miR-28-5p and was upregulated with miR-28-5p inhibitor treatment. (**E**–**G**) By qRT-PCR assay, RAP1B mRNA was not altered after overexpression or knockdown of miR-28-5p in A498 and ACHN cells. (**H**) Luciferase reporter assays were performed in A498 and ACHN cells by cotransfection of wild type RAP1B-3′-UTR vector or mutant RAP1B-3′-UTR vector and miR-28-5p mimics or negative control. The expression of miR-28-5p mimics significantly suppressed the luciferase activity of the wild type reporter but had minimal effect on the mutant reporter. ^*^P < 0.05; ^**^P < 0.01.

To further validate RAP1B as a legitimate target of miR-28-5p, we analyzed the expression of the endogenous RAP1B after transiently transfecting cells with miR-28-5p mimics or miR-28-5p inhibitor. The transient transfection efficiencies of miR-28-5p mimics and miR-28-5p inhibitor in renal carcinoma cell lines were detected by qRT-PCR (Supplementary Figure S2A and S2B). As anticipated, RAP1B protein levels in both A498 and ACHN cells were dramatically reduced by miR-28-5p mimics and significantly increased by miR-28-5p inhibitor (Figure 3A and 3B). To further examine miR-28-5p can inhibit RAP1B expression *in vitro*, we also stably overexpressed miR-28-5p by transfecting a lentiviral expressing vector of miR-28-5p into the above two cell lines. Stable ectopic expression of miR-28-5p in these cells was validated by qRT-PCR (Supplementary Figure S1C). The secretion of RAP1B protein was also drastically diminished by the lentiviral miR-28-5p expression vector to the same degree as by miR-28-5p mimics (Figure 3C and 3D). In contrast, RAP1B mRNA expression was not affected by overexpression or knockdown of miR-28-5p in A498 or ACHN cells (Figure 3E and 3F). Overexpression of plenti-miR-28-5p by a lentivirus did not affect RAP1B mRNA level in the above-mentioned cell lines either (Figure 3G). Taken together, these results demonstrated that miR-28-5p modulated the protein level, but not the mRNA level of RAP1B.

To confirm whether RAP1B is a direct and specific target of miR-28-5p, we employed a luciferase reporter carrying the presumed site in the 3′-UTR of RAP1B mRNA. A control construct was also generated in which the putative miR-28-5p target binding sequence was mutated (Figure 2B). The recombinant wild type RAP1B 3′-UTR constructs or mutant 3′-UTR constructs were transfected into human renal carcinoma cell lines A498 and ACHN together with miR-28-5p mimics or negative controls. As shown in the Figure 3H, as comparing with controls, expression of miR-28-5p mimics significantly suppressed the luciferase activity of the reporter, but had minimal effect on the mutant reporter, suggesting that miR-28-5p can bind directly to the RAP1B 3′-UTR. This indicates that RAP1B is a bona fide target of miR-28-5p.

### miR-28-5p suppresses renal carcinoma cell proliferation

**Figure 4:**
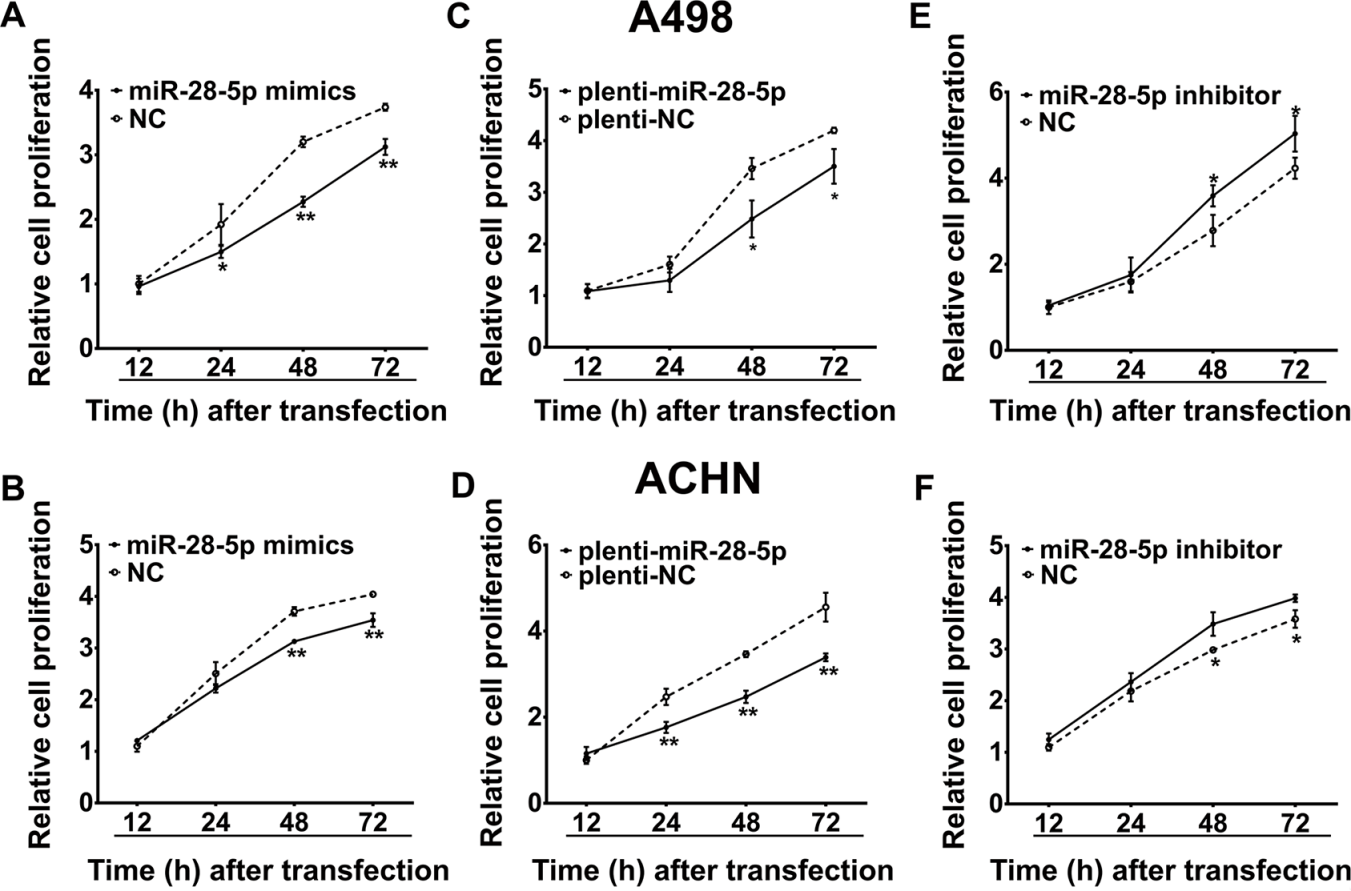
miR-28-5p suppresses renal carcinoma cell proliferation (**A**–**D**) By CCK-8 assay, the proliferation of A498 and ACHN cells was significantly inhibited by transient transfection with miR-28-5p mimics or stable transfection with plenti-miR-28-5p. (**E**, **F**) The proliferative abilities of A498 and ACHN cells were significantly promoted by transient transfection with miR-28-5p inhibitor. ^*^P < 0.05; ^**^P < 0.01.

To understand the biological effect of miR-28-5p downregulation on the proliferation and migration of renal carcinoma cells, miR-28-5p expression was manipulated *in vitro* and gain-of-function and loss-of-function analyses were performed. We first evaluated the effects of miR-28-5p on renal carcinoma cell proliferation using the Cell Counting Kit-8 (CCK-8) assay. As shown in Figure 4A and 4B, overexpression of miR-28-5p by transient transfection of miR-28-5p mimics significantly reduced the growth rate of A498 and ACHN cells. Similar results were found in these cell lines infected with the miR-28-5p overexpression lentivirus plenti-miR-28-5p as indicated in Figure 4C and 4D. In contrast, silencing of miR-28-5p expression significantly promoted the growth of A498 and ACHN cells (Figure 4E and 4F). To further confirm the role of miR-28-5p in renal carcinoma cell proliferation, we subsequently performed cell cycle analysis and colony formation assay when miR-28-5p was overexpressed or inhibited in A498 and ACHN cells, respectively. As shown in the Supplementary Figure S3, compared with the controls, A498 and ACHN cells transfected with miR-28-5p had a slightly higher percentage of cells in G1 phase and a lower percentage of cells in S phase, while inhibited miR-28-5p expressions had opposite effects, suggesting that miR-28-5p causes G1 arrest. In addition, overexpression of miR-28-5p significantly suppressed the colony formation, while the loss of miR-28-5p function obviously enhance the proliferation of RCC cell lines. Therefore, these *in vitro* results further confirmed that miR-28-5p had a biological effect on proliferation (Supplementary Figure S3).

### miR-28-5p represses renal carcinoma cell migration and invasion

**Figure 5:**
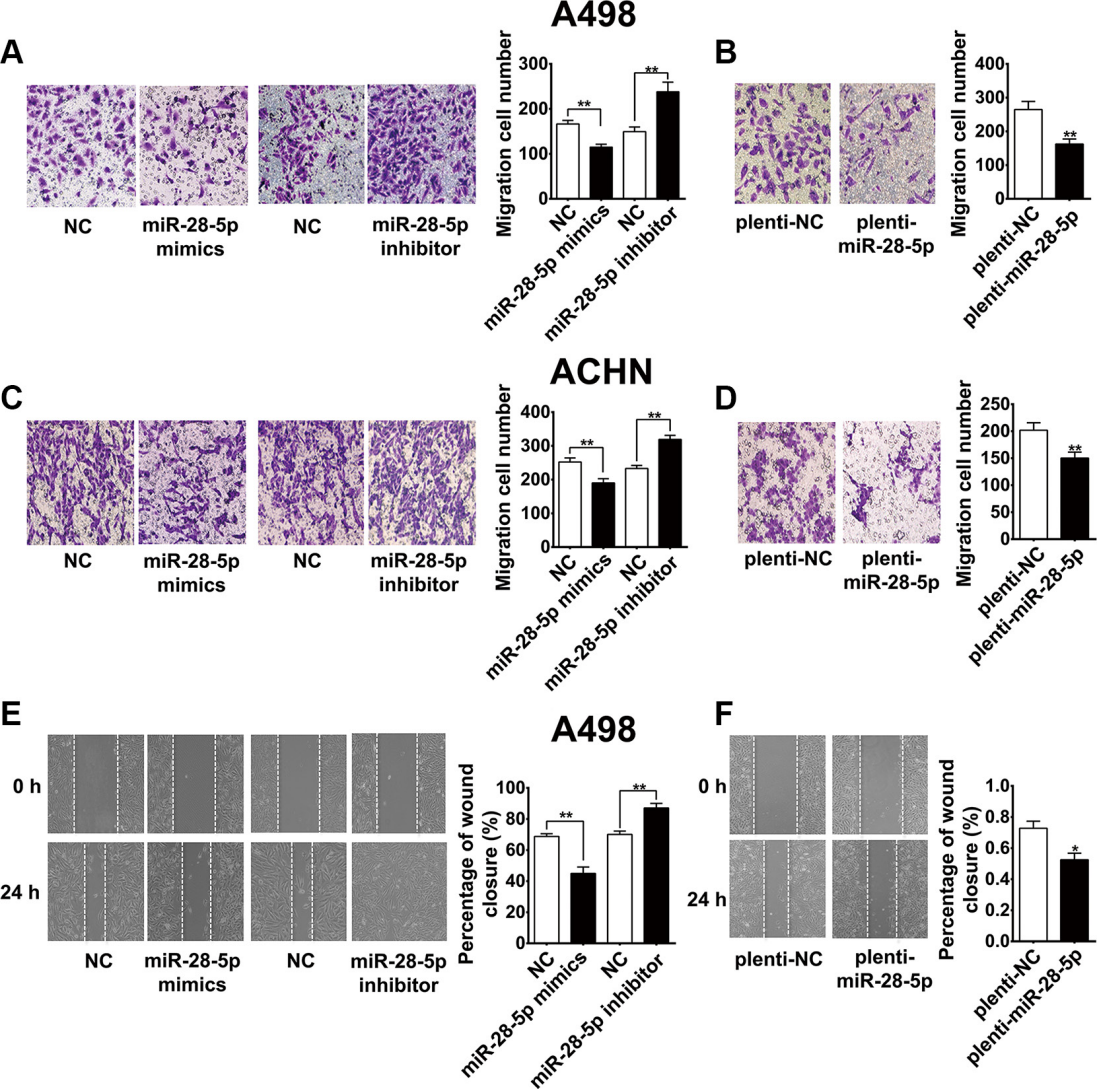
miR-28-5p represses renal carcinoma cell migration (**A**–**D**) Transwell migration assays using A498 and ACHN cells showed that miR-28-5p overexpression by transient transfection with miR-28-5p mimics or stable transfection with plenti-miR-28-5p both resulted in an increased number of relocated cells compared with vector control transfection, whereas miR-28-5p inhibitor-mediated knockdown of miR-28-5p markedly enhanced the migration of these cells compared with nontransfected counterparts. (**E**, **F**) Wound-healing assay showed that A498 cell mobility was reduced with miR-28-5p ectopic expression by miR-28-5p mimics or plenti-miR-28-5p, and was markedly increased with miR-28-5p inhibitor treatment.^*^P < 0.05; ^**^P < 0.01.

Next, we assessed the possible role of miR-28-5p in renal carcinoma cell migration. A transwell migration assay using A498 and ACHN cell lines showed that ectopic expression of miR-28-5p using a transient transfection strategy with miR-28-5p mimics or a stable lentiviral infection strategy with plenti-miR-28-5p decreased the number of invaded cells compared with that of the corresponding controls (Figure 5A–5D). In contrast, miR-28-5p inhibitor-mediated knockdown of miR-28-5p markedly enhanced renal carcinoma cell migration (Figure 5A and 5C). Furthermore, the wound-healing assay showed that the rate of A498 cell wound-healing was drastically reduced by miR-28-5p overexpression (Figure 5E and 5F), and it was markedly increased by miR-28-5p inhibitor treatment compared with treatment with scramble counterparts (Figure 5E). Collectively, our data suggest that miR-28-5p strongly contributes to the development of renal carcinoma migration.

**Figure 6:**
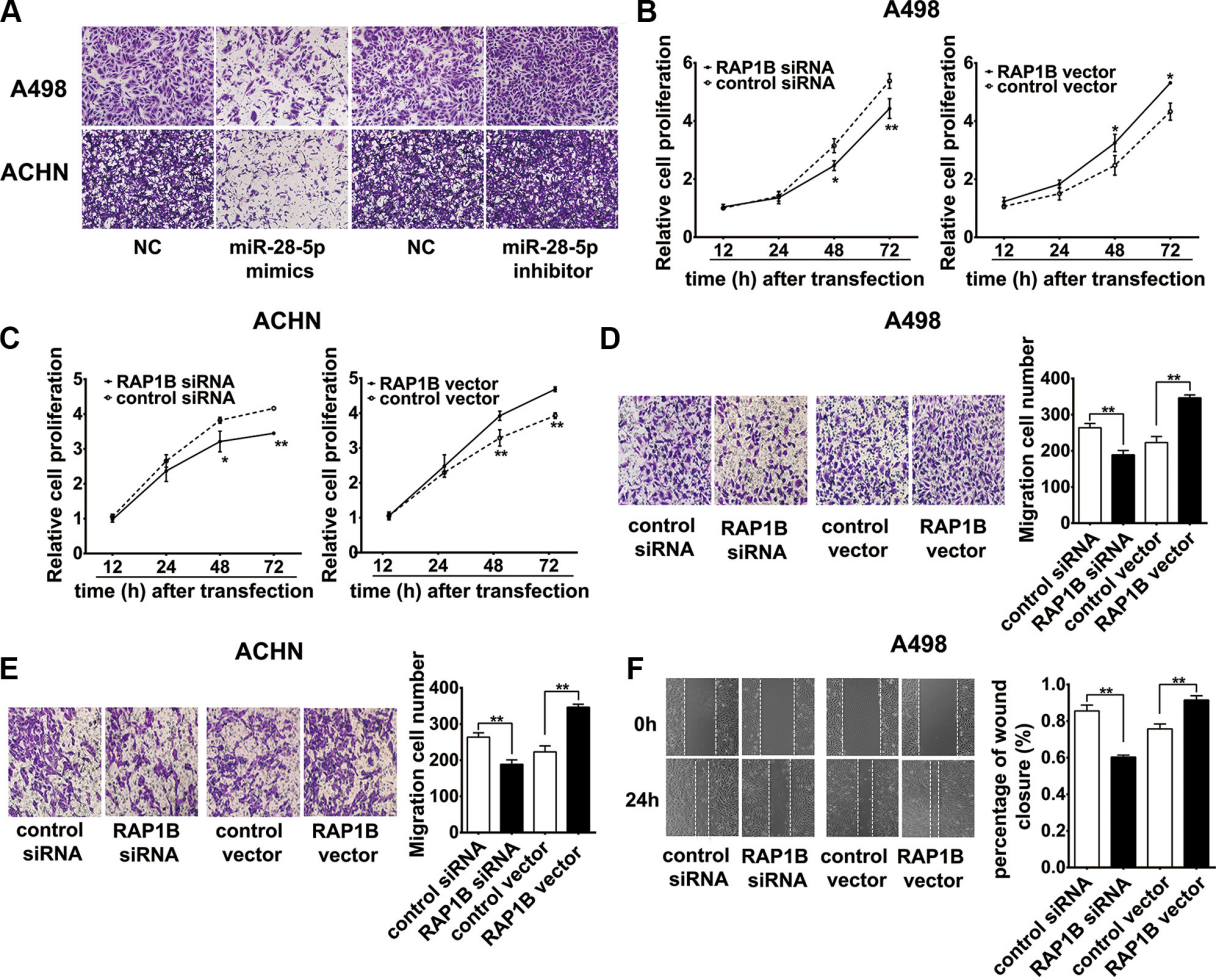
miR-28-5p represses renal carcinoma cell invasion and RAP1B promotes renal carcinoma cell proliferation and migration (**A**) Ectopic expression of miR-28-5p inhibited the invasion of A498 and ACHN cells *in vitro*. Compared with control, miR-28-5p attenuated cell invasion ability in A498 and ACHN cells by decreasing the cell numbers passing through the matrigel membrane. A representative experiment is shown. Microscopy images (×20) show the invasive cells on transwell assays and each representative experiment was performed in triplicate. (**B**, **C**) By CCK-8 assay, the proliferation of A498 and ACHN cells was significantly inhibited by the knockdown of RAP1B expression with RAP1B siRNA, while the overexpression of RAP1B by RAP1B vector markedly enhanced the growth rate of these cell lines. (**D**, **E**) By transwell migration assays, RNAi-mediated knockdown of RAP1B in A498 and ACHN cells decreased the number of migrated cells, and RAP1B overexpression had the opposite effect. (**F**) The wound-healing assays showed that RAP1B siRNA significantly weakened the migration capabilities of A498 cells, while overexpression of RAP1B markedly promoted cell migration. ^*^P < 0.05; ^**^P < 0.01.

We then examined the effect of miR-28-5p on tumor cell invasion. By observing cell numbers passing through the matrigel membrane, we found that ectopic expression of miR-28-5p could significantly inhibit cell invasion ability in A498 and ACHN cell lines compared with controls, while transfection of miR-28-5p inhibitor significantly increase the invading cell population. miR-28-5p attenuated cell invasion ability in A498 and ACHN cells (Figure 6A). These results demonstrated that miR-28-5p could attenuate cell invasion ability in renal carcinoma cells.

### RAP1B promotes renal carcinoma cell proliferation and migration *in vitro*

To confirm whether RAP1B acts as an oncoprotein in RCC, we examined the effects of RAP1B on renal carcinoma cell proliferation *in vitro*. We evaluated the effects of RAP1B on renal carcinoma cell proliferation using the CCK-8 assay after transfecting A498 and ACHN cells with synthesized specific small interfering RNAs (siRNAs) against RAP1B mRNA or a RAP1B–expression vector. The efficiency of the expression of RAP1B siRNAs and exogenous RAP1B was examined by western blotting (Supplementary Figure S4A–S4C). Of the three synthesized RAP1B siRNAs (RAP1B siRNA-1, RAP1B siRNA-2 and RAP1B siRNA-3), RAP1B-siRNA-2 exhibited the most inhibition of the expression of RAP1B protein and, therefore, was chosen for use in the subsequent studies (Supplementary Figure S4A). The inhibition of RAP1B expression markedly impeded the proliferation of A498 and ACHN cells, while the overexpression of RAP1B significantly increased the growth rate of A498 and ACHN cells (Figure 6B and 6C).

We furthermore assessed the possible role of RAP1B on the migratory capacity of renal carcinoma cells. Transwell migration assays showed that RAP1B inhibition significantly decreased the number of invaded cells for both the A498 and ACHN cell lines (Figure 6D and 6E), and RAP1B overexpression had the opposite effect (Figure 6D and 6E). The wound-healing assay demonstrated that RNAi-mediated knockdown of RAP1B significantly weakened A498 cell migration, while overexpression of RAP1B markedly promoted cell migration in A498 cells (Figure 6F). Collectively, our data strongly suggest that RAP1B and miR-28-5p have opposing effects on the regulation of renal carcinoma cell migration.

### miR-28-5p represses renal carcinoma cell proliferation and migration by directly targeting RAP1B

**Figure 7:**
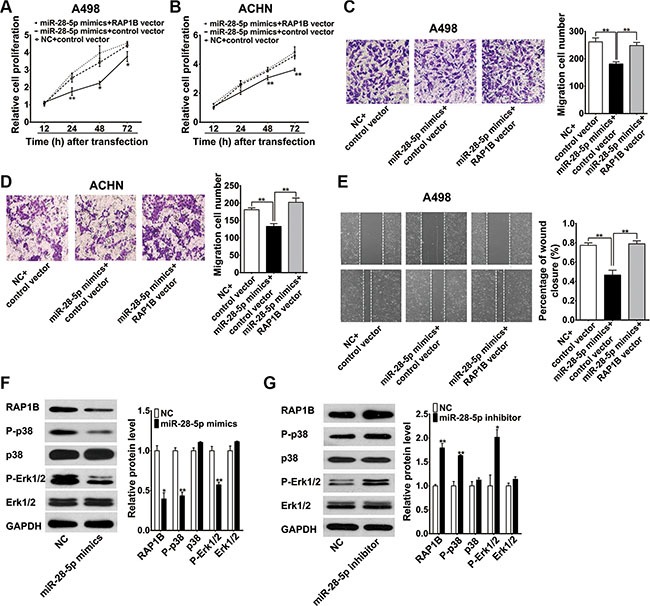
miR-28-5p represses renal carcinoma cell proliferation and migration by directly targeting RAP1B (**A**, **B**) CCK-8 assay indicated that the re-expression of RAP1B significantly reversed miR-28-5p-mediated inhibition of both A498 and ACHN cell growth. (**C**, **D**) The transwell migration assays demonstrated that miR-28-5p reduced approximately 30% of A498 and ACHN cell migration, and co-expression of RAP1B vector restored cell migration to the control level. (**E**) The wound-healing assay showed that re-expression of RAP1B significantly enhanced the cell motility of A498 cells. (**F**, **G**) Western blotting analysis showed that miR-28-5p overexpression in A498 cells decreased the phosphorylation levels of p38 and Erk1/2, whereas miR-28-5p inhibitor had opposite effects. ^*^P < 0.05; ^**^P < 0.01.

To further clarify whether the tumor suppression roles of miR-28-5p were dependent on RAP1B expression, we performed a series of functional restoration assays in A498 and ACHN cells. As shown in Figure 7A and 7B, re-expression of RAP1B significantly reversed miR-28-5p-leaded inhibition of A498 and ACHN cell proliferation. miR-28-5p reduced approximately 30% of A498 and ACHN cell migration, and co-expression of the RAP1B vector restored cell migration to the baseline level (Figure 7C and 7D). Moreover, re-expression of RAP1B significantly enhanced the wound-healing rate of A498 cells inhibited by miR-28-5p mimics (Figure 7E). These results indicate that re-expression of RAP1B significantly rescue the miR-28-5p -mediated inhibition of cell growth and migration, and the miR-28-5p/RAP1B signaling axis has an important role in the development and progression of RCC.

### miR-28-5p represses the phosphorylation MAPK signaling molecules by p38 and Erk1/2

The activation of two MAP kinases, p38 and Erk1/2, which are important regulators of endothelial migration and proliferation in the MAPK signaling pathway, has been shown to be decreased in RAP1B-deficient endothelial cells in response to vascular endothelial growth factor (VEGF) stimulation [29]. Thus, we tested whether the suppression of the expression of RAP1B resulted by miR-28-5p overexpression affects the activation of these two MAP kinases in renal carcinoma cells. A western blotting analysis showed that miR-28-5p overexpression in A498 cells not only suppressed the expression of RAP1B but also decreased the phosphorylation levels of p38 and Erk1/2. In contrast, knockdown of miR-28-5p with miR-28-5p inhibitor increased the expression of RAP1B and, at the same time, promoted the phosphorylation of p38 and Erk1/2; however, the total levels of p38 and Erk1/2 remained unchanged (Figure 7F and 7G). These data suggest that miR-28-5p may involve in repressing the phosphorylation of p38 and Erk1/2 through the inhibition of RAP1B, and further demonstrating that RAP1B is a key mediator of miR-28-5p function.

### miR-28-5p decreases the growth rate of renal carcinoma *in vivo*

**Figure 8:**
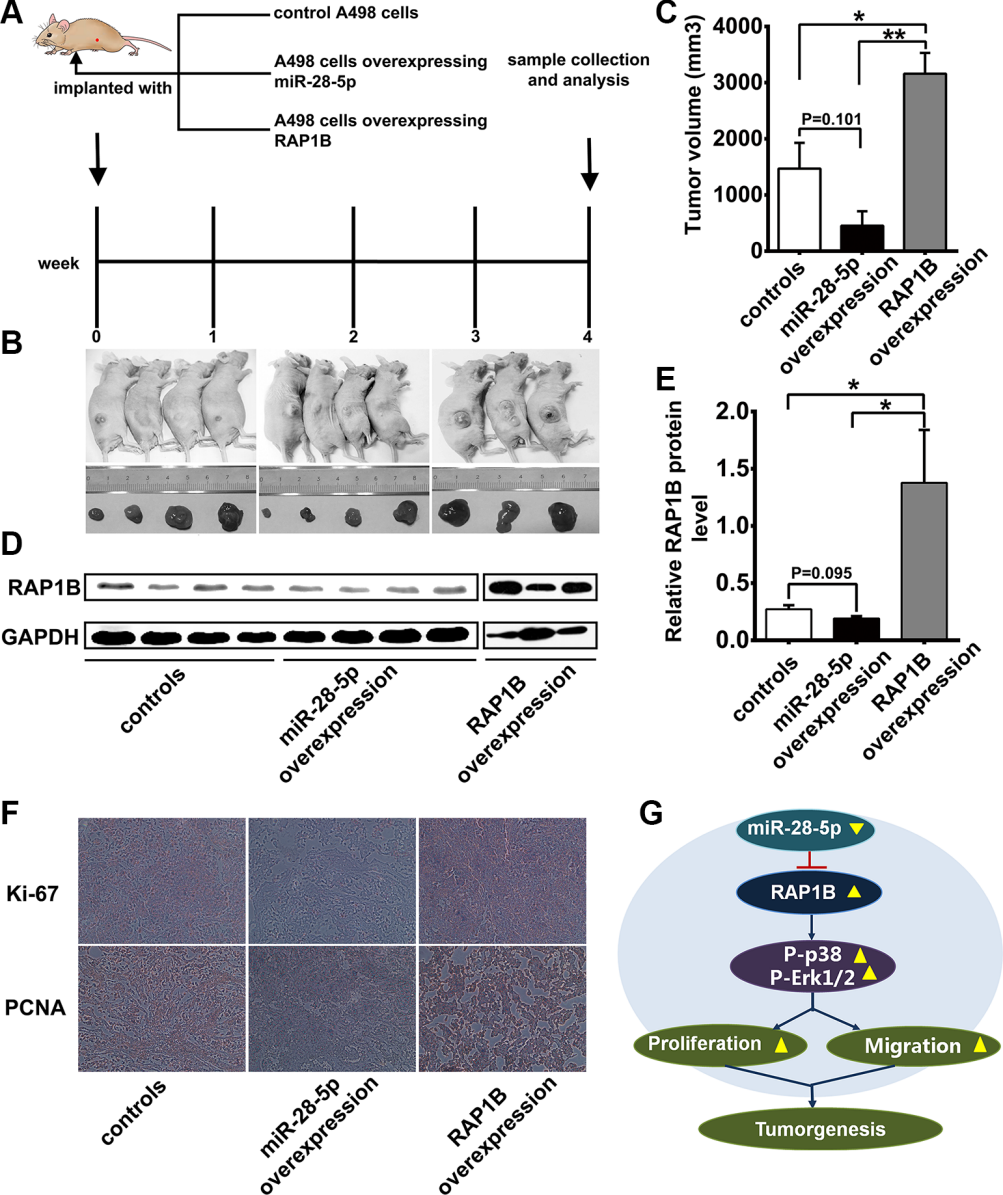
Effects of overexpression of miR-28-5p or RAP1B on the growth of renal cell carcinoma xenografts in mice (**A**) Flow chart of the experimental design. A498 cells were either infected with a lentiviral expression vector to express miR-28-5p or transfected with an RAP1B overexpression plasmid. A498 cells with increased miR-28-5p or RAP1B levels were then implanted subcutaneously into 4-week-old C57/BL6 mice. (**B**) The morphologic characteristics of tumors in mice inoculated with cells overexpressing miR-28-5p or RAP1B and control cells are shown. (**C**) The volume of xenograft tumor in the miR-28-5p-overexpression group, RAP1B-overexpressing group and negative control group. The volume of xenograft tumor in the miR-28-5p-overexpression group was lower than in the negative control group. (**D**, **E**) Western blotting analysis of RAP1B protein levels in the tumors from mice implanted with control cells, miR-28-5p-overexpressing cells, and RAP1B-overexpressing cells. (**F**) Representative experiment (×20) of proliferative activity assessed by anti-Ki-67 and anti-PCNA monoclonal antibody in the tumors from mice implanted with control cells, miR-28-5p-overexpressing cells, RAP1B-overexpressing cells. (**G**) Model to explain the function of miR-28-5p/RAP1B signaling axis in RCC. See the text for details.

Because our *in vitro* experiments revealed that miR-28-5p or RAP1B expression was associated with proliferative traits, we next examined the effect of miR-28-5p or RAP1B expression on tumor formation *in vivo*. We performed a tumor formation assay in a nude mouse model using A498 cells stably expressing miR-28-5p after lentiviral infection or transfected with a RAP1B plasmid to overexpress RAP1B, and then cells were implanted subcutaneously into mice (Figure 8A).

The mice were maintained under specific pathogen-free conditions at Jinling Hospital and were sacrificed 4 weeks later, and the tumors that had developed were weighed (Figure 8A). Unexpectedly, one mouse in RAP1B overexpression group was dead at the 3-week time point, thus only three tumors were obtained in this group (Figure 8B). Compared with the control mice, ectopic overexpression of miR-28-5p decreased the tumor volume of the nude mice (*P* = 0.101, Figure 8B–8C). In the meanwhile, we also observed that the average tumor volume was about two fold greater in the mice inoculated with A498 cells overexpressing RAP1B compared to the control mice (*P* < 0.01, Figure 8B–8C), and these results indicate that RAP1B acts as a promoter of renal carcinoma growth.

In addition, we also evaluated the expression level of RAP1B in xenograft tumors after ectopic overexpression of miR-28-5p or RAP1B. Western blotting showed that RAP1B expression in the *xenograft* tumors was slightly suppressed by overexpression of miR-28-5p (*P* = 0.095, Figure 8D–8E), while significantly increased by overexpression of RAP1B (*P* < 0.01, Figure 8D–8E) as compared with the control mice, and this suppression was mediated primarily through the suppression of the translation of RAP1B (Figure 8D–8E). To confirm the role of miR-28-5p/RAP1B axis in tumor cell proliferation or metastasis *in vivo*, we performed the immunohistochemistry analysis to assess the proliferative activity by using anti-Ki-67 and anti-PCNA monoclonal antibody in the tumors from mice implanted with control cells, miR-28-5p-overexpressing cells and RAP1B-overexpressing cells. As shown in Figure 8F, the cell proliferation rate, as measured by the ratio of Ki-67 and PCNA positive tumor cells, were increased in tumors from the RAP1B-overexpressing group and decreased in tumors from the miR-28-5p-overexpressing group (Figure 8F).

Taken together, these results combined with the results of the *in vitro* assays, which confirmed the tumor suppressor role of miR-28-5p in renal carcinoma tumorigenesis through the targeting of RAP1B.

## DISCUSSION

A review of the existing literature shows that miR-28-5p is aberrantly expressed in several types of tumors and cancer cell lines; however, only a few studies have analyzed miR-28-5p function in cancer. Girardot *et al*. observed miR-28 overexpression in the platelets of patients with BCR-ABL negative myeloproliferative neoplasms, and they identified MPL, an important regulator for megakaryocyte differentiation, to be the main target of miR-28 [33]. In another study, miR-28-5p was defined as a critical regulator of Mad2 translation and the function of the mitotic checkpoint in VHL-associated cancers [34]. However, contradictory roles for miR-28 have been reported in CRC. Almeida MI *et al*. found that overexpression of miR-28-5p reduced CRC cell proliferation, migration and invasion *in vitro* and inhibited tumor growth *in vivo* [18]. These data suggest complex roles for miR-28-5p in multiple types of tumors. Our previous study observed that miR-28-5p was markedly reduced in the sera of patients with RCC compared that of normal controls [17]. This suggests that miR-28-5p might be involved in the pathogenesis of RCC. The present work was the first to show a significant decrease in miR-28-5p expression in RCC tumors, and this was true for the majority of samples tested. Through *in vitro* and *in vivo* miR-28-5p gain-of-function and loss-of-function studies, we further confirmed that miR-28-5p acted as a tumor suppressor in RCC. In combination with our previous results, our current findings suggest that miR-28-5p has an antitumorigenic role in RCC and, therefore, has potential as a novel biomarker and therapeutic agent for RCC.

To explore the molecular mechanisms by which miR-28-5p affects RCC development, we predicted miR-28-5p target genes in RCC using the TargetScan [30], miRanda [31] and PicTar databases [32]. All three databases suggested that RAP1B is a miR-28-5p target gene, and we focused on this Ras-related GTPase for further study because it is an oncogene in a variety of tumors. Recently, some studies have documented RAP1B regulation at the post-transcriptional level by some tumor suppressor miRNAs [19, 21–23, 35]. However, the biological function of RAP1B in RCC and, in particular, the regulatory mechanism of RAP1B in RCC, has not been previously reported. Our study is the first to show that RAP1B is upregulated in RCC tumor samples and several renal carcinoma cell lines. More importantly, we found an inverse correlation between RAP1B protein expression and miR-28-5p levels in RCC tissues. Transfection of miR-28-5p mimics inhibited RAP1B protein expression in renal carcinoma cells, and transfection of miR-28-5p inhibitor promoted RAP1B protein expression. Functional studies showed that RAP1B has an opposite effect to that of miR-28-5p in the regulation of renal carcinoma cell proliferation and migration *in vitro* and also has an opposite effect to that of miR-28-5p in xenograft tumor growth *in vivo*. Furthermore, in our cell based studies, re-expression of RAP1B almost completely reversed the miR-28-5p-imposed inhibition on proliferation and migration. It has been demonstrated the regulation of RAP1B in proangiogenic signaling in endothelial cells. The activation of 2 MAP kinases, p38 MAPK and p42/44 ERK, which are important regulators of endothelial migration and proliferation, was decreased in RAP1B-deficient endothelial cells in response to VEGF stimulation [29]. Our investigation of the downstream effects of the miR-28-5p/RAP1B signaling axis showed that miR-28-5p may inhibit the phosphorylation of MAPK signaling molecules by p38 and Erk1/2 through the downregulation of RAP1B expression. These data reveal that miR-28-5p represses renal carcinoma cell proliferation and migration by targeting RAP1B, which we believe to be a novel function and mechanism of miR-28-5p and RAP1B in RCC.

An *in silico* search using three miRNA target databases identified seven genes as potential miR-28-5p targets. In this study, we focused on RAP1B and demonstrated that it was a functional target gene of miR-28-5p. However, it is possible that other potential targets of miR-28-5p in addition to RAP1B may be involved in the regulation of renal carcinoma cell proliferation and migration. Further studies should be performed to determine whether other targets of miR-28-5p and the signaling pathways they act on are involved in the mediation of RCC development and progression by miR-28-5p.

Based on our results, we propose the following model for the miR-28-5p/RAP1B signaling axis in RCC progression (Figure 8G). In RCC tissues and renal carcinoma cells, miR-28-5p is expressed at a low level and RAP1B is expressed at a high level. miR-28-5p inhibits the tumorigenesis of RCC by directly downregulating RAP1B and influencing the activation of two MAP kinases in the MAPK signaling pathway, p38 and Erk1/2. The newly identified miR-28-5p/RAP1B axis provides further insight into the pathogenesis of RCC and opens new avenues for future RCC therapies.

## MATERIALS AND METHODS

### Cell culture

The human renal carcinoma cell lines Caki-1, ACHN, immortalized primary human proximal tubular cell HK-2 and human embryonic kidney (HEK)-293T were purchased from Shanghai Cell Bank, Chinese Academy of Sciences (Shanghai, China) and cultured in the following media: McCoy's 5A (Sigma-Aldrich, St Louis, MO, USA), MEM-NEAA (GBICO, Beijing, China), DMEM/F12 (GBICO) and DMEM medium respectively. The A498 cell line was purchased from Cell Resource Center, IBMS, CAMSI/PUMC and cultured in MEM-NEAA medium.

### Tissue samples

The tissue collection and analyses were approved by the Ethics Committee of Jinling Hospital of Nanjing University, and written informed consent was obtained from all participants. Thirty-three pairs of RCC tissues and their matched non-tumorous adjacent tissues were obtained from patients undergoing surgery for RCC at the Jinling Hospital. All patients underwent a tumorectomy prior to the receipt of any adjunctive therapy. Pathology specimens from all patients were centrally reviewed and the definitive tumor stage was established on the basis of operative findings according to the WHO's TNM classification system for RCC [36]. All tissues were immediately snap-frozen in liquid nitrogen and stored at – 80°C until analysis.

### RNA isolation and qRT–PCR

Total RNA was extracted from tissues or cells with TRIzol reagent (Invitrogen, Carlsbad, CA, USA) following the manufacturer's protocol. miR-28-5p levels were determined by qRT–PCR using TaqMan assay kits (Applied Biosystems, Foster City, CA, USA) with the U6 sRNA as an internal reference. RAP1B mRNA was detected by qRT–PCR using a SYBR Premix E × Taq Reverse Transcription–PCR kit (Takara, Dalian, China), and values were normalized to GAPDH. The primers specific for RAP1B and GAPDH were as follows: RAP1B (sense) 5′-GTGAATCCCTTGCT TGCTCAT-3′ and RAP1B (anti-sense) 5′-AATACTGTGGCTCCCTGTTGG-3′; and GAPDH (sense): 5′-GATATTGTTGCCATCAATGAC-3′ and GAPDH (anti-sense): 5′-TTGATTTTGGAGGGAT CTCG-3′. The reactions were performed using an ABI PRISM 7900 Sequence Detection System (Applied Biosystems).

### Vector and plasmid construction and transfection

miR-28-5p mimics and its negative control (NC) mimics-scramble, miR-28-5p inhibitor and its NC inhibitor-scramble, and the miR-28-5p mutant were obtained from GenePharma (Shanghai, China), and lentivirus to overexpress miR-28-5p was purchased from Invitrogen. RAP1B siRNAs were obtained from RiboBio Co., Ltd. (Guangzhou, China). Their sequences were as follows: miR-28-5p mimics (sense) 5′-AAGGAGCUCACAGUCUAUUGAG-3′ and miR-28-5p mimics (anti-sense) 3′-C AAUAAGACUGUGAG CUCCUUU-5′; mimics-scramble (sense) 5′-UUCUCCG AACGUGUCACGUTT-3′ and mimic-scramble (anti-sense) 3′-ACGUGACACGUU CGGAGAATT-5′; miR-28-5p inhibitor (sense) 5′-CUCAAUAGACUGUAGCUCCU U-3′; inhibitor-scramble (sense) 5′-CAGUACUUU UGUGUAGUACAA-3′; RAP1B- siRNA1 (sense) 5′-GUGAGUAUAAGCUAGUCGUdTdT-3′; siRNA2 (sense) 5′-AC CUAGUGCGGCAAAUUAAdTdT-3′; and siRNA3 (sense) 5′-UGUUGGUAAUAU UGACdTdT-3′. The miR-28-5p mutant was constructed by replacing the RAP1B binding site with its complimentary sequence. A mammalian expression plasmid encoding the human RAP1B open reading frame (pReceiver-M02-RAP1B) was purchased from GeneCopoeia (Germantown, MD, USA). An empty plasmid served as the negative control. All constructs were verified by sequencing.

### Target prediction and luciferase reporter assays

The miRNA targets were predicted using the miRanda [30], TargetScan [31] and PicTar [32] databases. Luciferase reporter constructs containing regions of the RAP1B 3′-UTR with the indicated miR-28-5p target sites or mutant sites were cloned into a p-MIR-reporter plasmid (Ambion). A498 and ACHN cells were seeded in triplicate in 6-well plates and allowed to settle for 12 hours. Indicated plasmids and equal amounts (100 pmol) of miR-28-5p mimics, miR-28-5p inhibitor or NC RNA were transfected into the cells using Lipofectamine 2000 Reagent (Life Technologies). Twenty-four hours after transfection, firefly activity was measured using the luciferase assay kit (Promega, Madison, WI, USA).

### Immunohistochemical staining

Formalin-fixed, paraffin-embedded RCC tissues were subjected to standard IHC. A rabbit monoclonal anti-RAP1B antibody (Cell Signaling Tech., Danvers, MA, USA) was used in this study. The results of immunostaining were scored blindly with no information of the clinical data by Dr. Caiyun Wu and who were guided by a pathologist (Dr. Qiuyuan Xia at Department of Pathology, Jinling Hospital). The scoring was based on the intensity and extent of staining and was evaluated according to the following histological scoring methods as previously described [37, 38]. Briefly, the mean proportion of staining cells per specimen was determined semi-quantitatively of the examined cells. Staining intensity was graded as follows: 0 (negative), 1 (weakly positive), 2 (moderately positive), and 3 (strongly positive). The histological score for each specimen was computed by the formula: H-score = Percentage of stained cells × Intensity score. Tumor section slides were subjected to standard IHC analysis using Ki-67 staining and PCNA staining (Cell Signaling Tech., Danvers, MA, USA) according to the manufacturer's instructions.

### Western blotting analysis

The antibodies used for western blotting were as following: rabbit monoclonal anti-RAP1B antibody, anti-p-p38 antibody, anti-Erk1/2 antibody and anti-p-Erk1/2 antibody, and rabbit polyclonal anti-p38 antibody (all from Cell Signaling Tech.); mouse monoclonal anti-GAPDH antibody (Santa Cruz Biotechnology, Santa Cruz, CA, USA).

### Cell proliferation, migration and invasion analysis

Cell proliferation was evaluated using a WST-8 Cell Counting Kit-8 (CCK-8, Beyotime Biotech., Shanghai, China). A498 and ACHN cells were seeded in 96-well culture plates at 1 × 10^4^ cells/well (A498) or 2 × 10^4^ cells/well (ACHN), transfected with the indicated miR-28-5p mimics, miR-28-5p inhibitor, plenti-miR-28-5p, RAP1B vector, RAP1B siRNA or negative controls, and incubated for 12, 24, 48 and 72 h. Cell cycle analysis was conducted using flow cytometry after overexpressing or inhibiting miR-28-5p expression through transient transfection following 48 h culture. For colony formation assay, RCC cells were seeded in 2 ml medium per well in the 6-well culture plates (100 cells per well for A498 and ACHN). After cultured for 14 days, cells were fixed with paraformaldehyde and stained with crystal violet.

A wound-healing assay was performed to assess cell migration. An artificial wound was created 24 h after transfection using a 10 μl pipette tip. The A498 and ACHN cells were then cultured with 2% fetal bovine serum for 20 h. To visualize the migrated cells and wound healing, images were taken at 0 and 20 h.

The transwell migration was tested in a Transwell Boyden Chamber (6.5 mm, Costar, USA). Cells were harvested 48 h after transfection, and A498 and ACHN cells were seeded at a density of 5 × 10^5^ cells/well on the upper chamber with serum-free MEM-NEAA medium. Cells were allowed to invade the lower compartment for 17 h. Each experiment was performed in triplicate and repeated twice.

Cell invasion was evaluated using a Boyden chamber system with a polycarbonate membrane (8-μm pore size; Corning, New York, NY). For cell invasion assay, RCC cells (5 × 10^4^) were transfected with miR-28-5p mimics, miR-28-5p inhibitor or negative controls, and cell invasion was performed by matrigel invasion assay. Cells in the upper compartment of the chamber were suspended in serum-free medium, and the lower chamber contained medium supplemented with 10% fetal bovine serum. After incubated for 48 h, cells passing through the matrigel membrane were fixed and stained with crystal violet, followed by counting from three random microscopic fields.

### Tumor xenografts in nude mice

Four-week-old male nude mice were purchased from the Model Animal Research Center of Nanjing University (Nanjing, China). After 4 days of acclimatization, a total of 7 × 10^6^ A498 cells stably transfected with either the miR-28-5p overexpression lentiviral vector, RAP1B overexpression vector or control were injected subcutaneously into the right inside of the groin of each mouse (4 mice per group). The mice were sacrificed 4-week after seeding the tumor cells. The tumor weights were then determined and their ellipsoid volumes were calculated using the following formula: Volume = π/6 × (length) × (width) × (height). All animal care and handling procedures were performed in accordance with the National Institutes of Health's Guide for the Care and Use of Laboratory Animals and were approved by the Institutional Review Board of Jinling hospital (Nanjing, China).

### Statistical analysis

Each experiment was conducted at least three times. The results are presented as means ± s.d. The statistical significance of the differences was evaluated using Student's *t-test*. Comparisons between more than two groups were conducted using one-way analysis of variance (ANOVA), and the differences between groups were subsequently determined by the Fisher LSD test, when appropriate. Spearman's rank correlation analysis was used to examine the correlation between the relative expressions of miR-28-5p and RAP1B. A *P value* < 0.05 was considered statistically significant. SPSS version 13.0 (SPSS Inc., Chicago, IL, USA) software was used for all data analyses.

## SUPPLEMENTARY MATERIALS TABLES


