# Supplementary material for: miR-28-5p acts as a tumor suppressor in renal cell carcinoma for multiple antitumor effects by targeting RAP1B
Source: Oncotarget. 2016 Oct 7;7(45):73888–902. doi: 10.18632/oncotarget.12516 (PMC5342021; doi:10.18632/oncotarget.12516)
Supplement: Supplementary file 1 [file oncotarget-07-73888-s001.pdf]

miR-28-5p acts as a tumor suppressor in renal cell carcinoma for multiple antitumor effects by targeting RAP1B

Supplementary Materials

Supplementary Table S1: Demographic and clinical features of the RCC patients

Variable	RCC patients (*n* = 33)
**Age, years[a]**	
Mean ± s.d.	54 ± 13
Median (range)	50 (28–79)
**Sex, no (%)**	
Male	21 (63.6)
Female	12 (36.4)
**Smoking status, no. (%)**	
Ever and current	3 (9.1)
Never	30 (90.9)
**Alcohol consumption, no. (%)**	
Ever and current	3 (9.1)
Never	30 (90.9)
**Family history of RCC, no (%)**	
Yes	0
No	33 (100)
**Histological types, no (%)**	
Clear cell carcinoma	29 (87.9)
Chromophobe cell carcinoma<br>Gene fusion type of renal cell carcinoma	4 (12.1)
**TNM stage, no (%)**	
I–II	24 (72.7)
III–IV	6 (18.2)
Unknown	3 (9.1)
**Metastasis**	
Yes	2 (6.1)
No	32 (93.9)

Supplementary Table S2: The clinical information and relative expression of miR-28-5p of the 20 paired tissue specimens used for immunohistochemistry (IHC) analysis

ID number	Age	Gender	Histological types	TNM stage	Metastasis	Relative miR-28-5p fold change
P25	50	Male	Clear cell carcinoma	II-III	No	1.26
P32	70	Female	Clear cell carcinoma	I	No	2.11
P17	55	Female	Clear cell carcinoma	I	No	0.46
P20	49	Male	Clear cell carcinoma	I-II	No	0.91
P29	49	Male	Clear cell carcinoma	I-II	No	1.54
P19	22	Female	Gene fusion type of renal cell carcinoma	Unknown	No	0.86
P12	41	Female	Chromophobe cell carcinoma	Unknown	No	0.27
P33	40	Female	Clear cell carcinoma	II	No	2.18
P10	67	Male	Clear cell carcinoma	III	No	0.27
P7	47	Male	Chromophobe cell carcinoma	Unknown	No	0.16
P4	67	Female	Clear cell carcinoma	II	No	0.07
P3	72	Female	Clear cell carcinoma	II	No	0.04
P14	51	Female	Clear cell carcinoma	I-II	No	0.29
P22	59	Female	Clear cell carcinoma	II	No	1.15
P11	50	Female	Clear cell carcinoma	II	No	0.27
P13	47	Male	Chromophobe cell carcinoma	I	No	0.29
P27	41	Male	Clear cell carcinoma	IV	No	1.48
P21	77	Male	Clear cell carcinoma	II	No	0.95
P1	45	Female	Clear cell carcinoma	II-III	No	0.02
P9	63	Male	Clear cell carcinoma	I	No	0.23

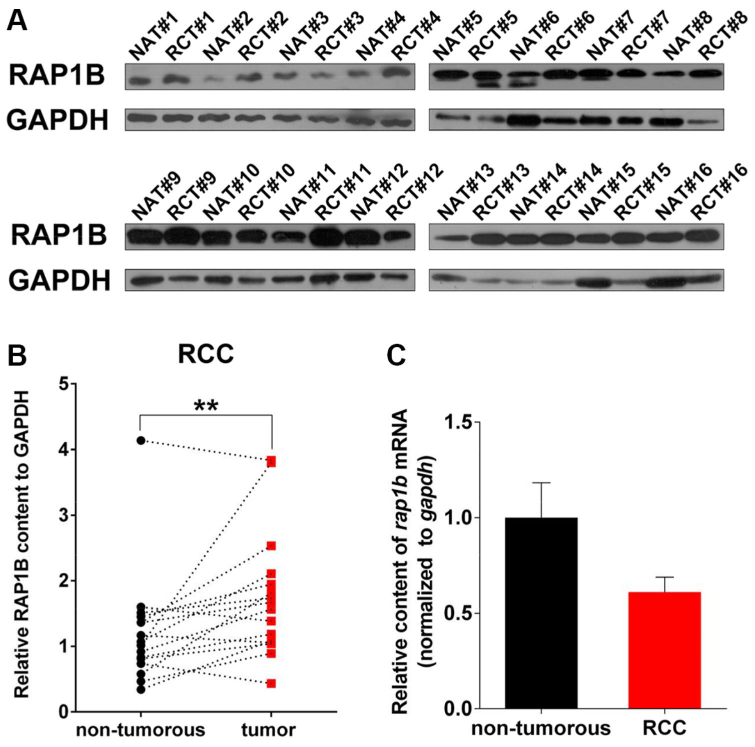

Supplementary Figure S1: The RAP1B protein and mRNA expression levels in RCC tissue samples and corresponding adjacent non-tumorous samples. (**A**) The expression level of RAP1B protein in tissue samples were measured using western blotting (**B**) and normalized against to GAPDH. (**C**) The relative content of RAP1B mRNA to GAPDH were assessed using qRT-PCR. The data were analyzed using the ΔΔCt approach [$2^{-\Delta Ct}$].

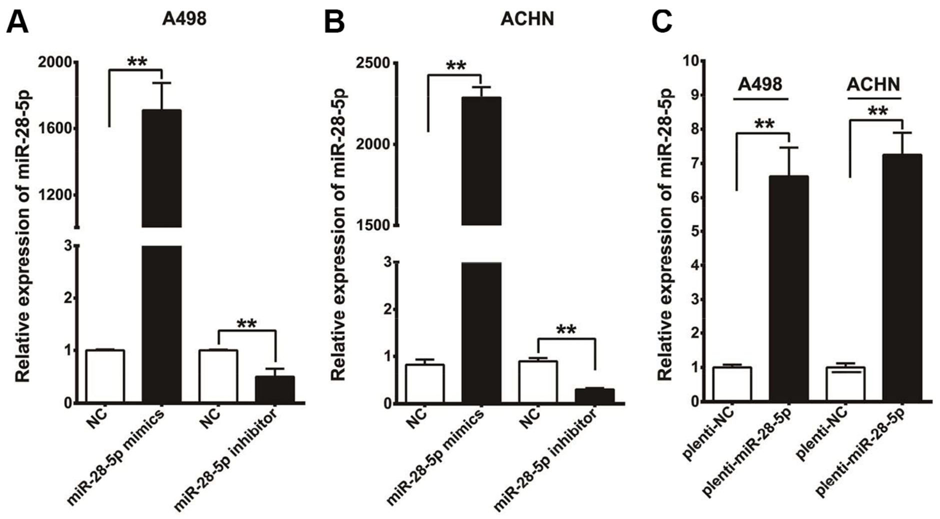

Supplementary Figure S2: Transfection efficiency of miR-28-5p mimics, miR-28-5p inhibitor and plenti-miR-28-5p in renal carcinoma cell lines. (**A**, **B**) qRT–PCR results of miR-28-5p expression levels after transient transfection of miR-28-5p mimics or miR-28-5p inhibitor into renal carcinoma cell lines A498 and ACHN. (**C**) qRT–PCR results of miR-28-5p expression levels after stable overexpression of miR-28-5p by transfecting plenti-miR-28-5p into A498 and ACHN cells. **$P < 0.01$.

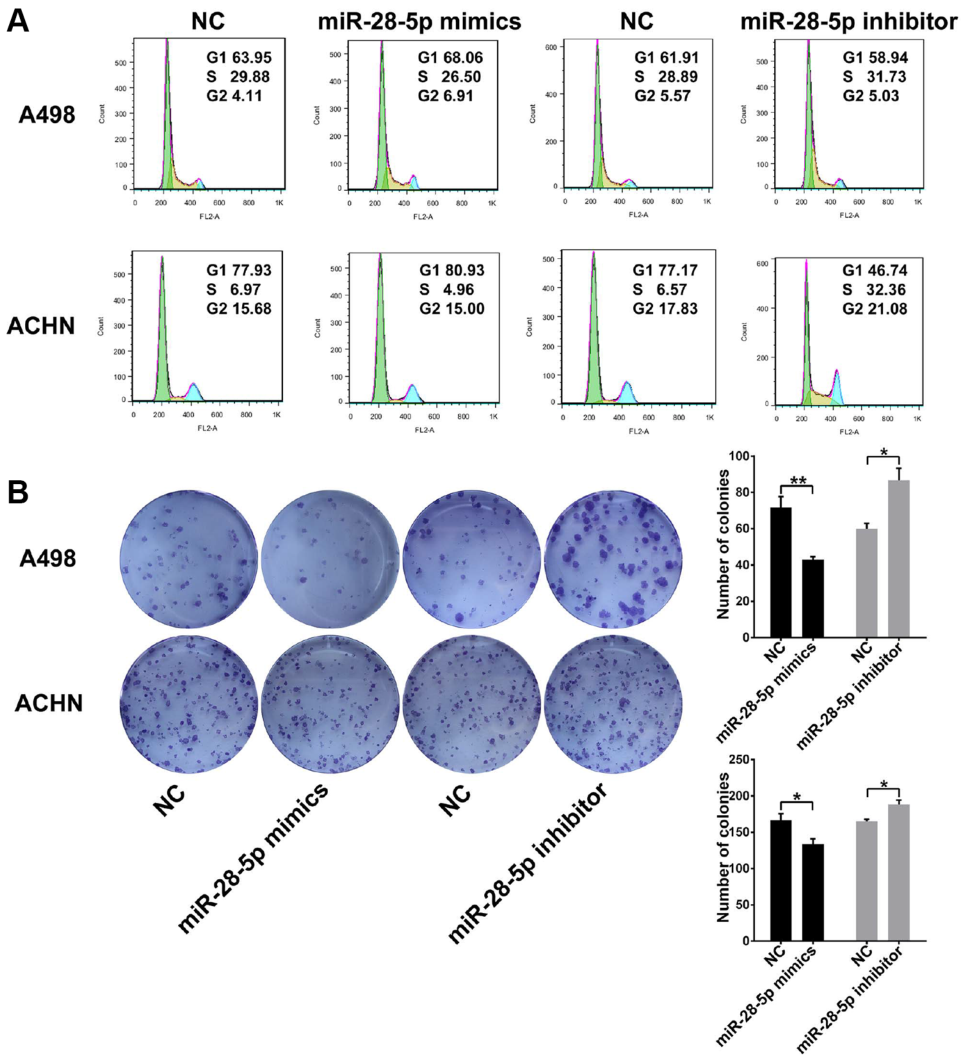

Supplementary Figure S3: Biological effects of miR-28-5p in cell cycle and colony formation *in vitro*. (**A**) Fluorescent-activated cell sorting analysis 48 hours post-transfection with Negative control, miR-28-5p mimics and miR-28-5p inhibitor in A498 and ACHN cell lines. Representative experiment was performed in triplicate. (**B**) Representative experiment of the biological effect of Negative control, miR-28-5p mimics and miR-28-5p inhibitor in A498 and ACHN cell lines. Values represent the mean of 3 replicates ± standard error (**P* < 0.05, ***P* < 0.01, Student *t* test).

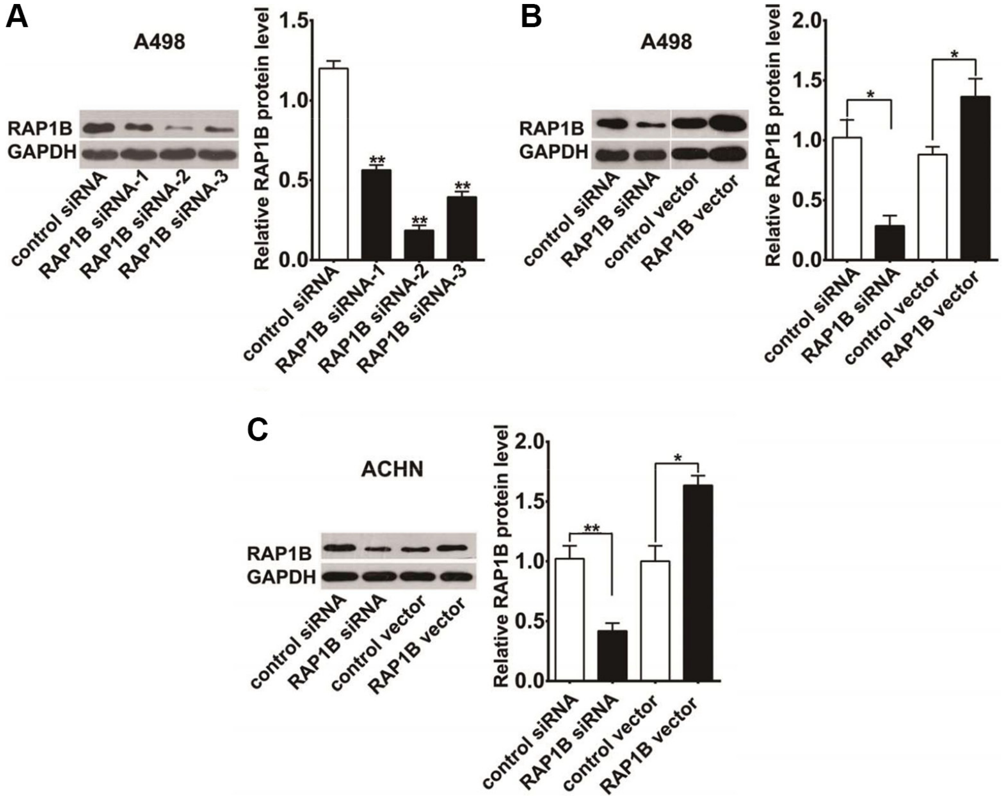

Supplementary Figure S4: The efficiency of RAP1B siRNAs and RAP1B vector on the expression of RAP1B in renal carcinoma cell lines. (**A**) By western blotting analysis, RAP1B protein was significantly downregulated in A498 cells transfected with siRNAs targeting RAP1B (RAP1B siRNA-1, RAP1B siRNA-2 and RAP1B siRNA-3). (**B**, **C**) By western blotting analysis, RAP1B protein was significantly downregulated in A498 and ACHN cells transfected with RAP1B siRNA-2, whereas markedly upregulated in A498 and ACHN cells transfected with RAP1B vector. $*P < 0.05$, $**P < 0.01$.
